# Risk factors for unintentional poisoning in children aged 1–3 years in NSW Australia: a case–control study

**DOI:** 10.1186/1471-2431-13-88

**Published:** 2013-05-24

**Authors:** Marcia Schmertmann, Ann Williamson, Deborah Black, Leigh Wilson

**Affiliations:** 1 School of Public Health and Community Medicine, University of New South Wales, Sydney, Australia; 2 Faculty of Science, University of New South Wales, Sydney, Australia; 3 Faculty of Health Sciences, University of Sydney, Sydney, Australia

**Keywords:** Child, Poisoning, Risk factors, Odds ratios

## Abstract

**Background:**

Unintentional poisoning in young children is an important public health issue. Age pattern studies have demonstrated that children aged 1–3 years have the highest levels of poisoning risk among children aged 0–4 years, yet little research has been conducted regarding risk factors specific to this three-year age group and the methodologies employed varied greatly. The purpose of the current study is to investigate a broad range of potential risk factors for unintentional poisoning in children aged 1–3 years using appropriate methodologies.

**Methods:**

Four groups of children, one case group (children who had experienced a poisoning event) and three control groups (children who had been ‘injured’, ‘sick’ or who were ‘healthy’), and their mothers (mother-child dyads) were enrolled into a case–control study. All mother-child dyads participated in a 1.5-hour child developmental screening and observation, with mothers responding to a series of questionnaires at home. Data were analysed as three case–control pairs with multivariate analyses used to control for age and sex differences between child cases and controls.

**Results:**

Five risk factors were included in the final multivariate models for one or more case–control pairs. All three models found that children whose mothers used more positive control in their interactions during a structured task had higher odds of poisoning. Two models showed that maternal psychiatric distress increased poisoning risk (poisoning-injury and poisoning-healthy). Individual models identified the following variables as risk factors: less proximal maternal supervision during risk taking activities (poisoning-injury), medicinal substances stored in more accessible locations in bathrooms (poisoning-sick) and lower total parenting stress (poisoning-healthy).

**Conclusions:**

The findings of this study indicate that the nature of the caregiver-child relationship and caregiver attributes play an important role in influencing poisoning risk. Further research is warranted to explore the link between caregiver-child relationships and unintentional poisoning risk. Caregiver education should focus on the benefits of close interaction with their child as a prevention measure.

## Background

Unintentional childhood poisoning causes significant morbidity and mortality in children throughout the world [1]. In New South Wales (NSW) Australia, unintentional poisoning mortality rates are very low across the childhood years overall [2], yet unintentional poisoning remains an ongoing public health problem for young children (i.e., ages 0–4 years) in particular. In 2010, the NSW Poisons Information Centre (PIC) received over 23,500 phone calls originating in NSW regarding unintentional poisoning in children aged 0–4 years (personal communication, J. Brown, NSW PIC, 27th Jan 2012). Emergency department data regarding unintentional childhood poisoning in NSW are not collected at a population level [3] but approximately 8,500 children aged 0–4 years were hospitalised for unintentional poisoning from 1994 to 2005 [4].

Unintentional poisoning statistics often group young children together but children aged 0–4 years do not constitute a homogenous group in terms of poisoning risk. An analysis of unintentional poisoning hospitalisations by single year of age showed that children aged 1–3 years had statistically significant higher hospitalisation rates compared to children aged <1 year and children aged 4 years [4]. Studies conducted on young children in other developed countries have reported similar age patterns for unintentional poisoning. These studies used hospital data (presentations and admissions) and PIC data, and reported frequencies [5-7], rates [8-11] and sometimes both frequencies and rates [12,13]. The findings of these studies demonstrate that children aged 1–3 years experience the highest levels of unintentional poisoning risk among children aged 0–4 years.

Young children also exhibit different age patterns regarding the type of substance causing unintentional poisoning. A NSW study showed that the odds of poisoning by medicinal substances compared to non-medicinal substances changed with age (in three-month intervals) [14]. Younger children were more likely to be poisoned by non-medicinal or household substances than older children. Other authors have also noted age patterns by substance type [8,12,15-19]. These findings indicate that young children are vulnerable to unintentional poisoning by different types of substances as they age.

Several authors have attributed these age patterns for unintentional poisoning to child development [4,10,14,16,20-22]. Specifically, young children differ widely in their level of physical, cognitive and self-regulatory development [23] and these differences are likely to result in different levels of poisoning exposure for young children due to the way they interact with hazards in their environment [10]. For example, young children who are able to explore their environment require greater supervision and more protective storage practices for poisons than those who are less mobile. However, studies have shown that supervision decisions and safety practices can be influenced by caregiver perceptions of the child’s developmental level [24-28]. Hence, inaccurate perception may result in inadequate supervision and poisons storage practices and increase a young child’s poisons exposure.

Previous studies have indicated that young children aged 1–3 years are the most vulnerable to unintentional poisoning and that factors, such as type of substance, child development, supervision and safety measures, may contribute to poisoning risk. In Australia, the state of NSW has the largest population of children aged 1–3 years, comprising an estimated 32.5 percent of all Australian children in this age group identified in the 2006 Census [29]. Therefore, it is important that risk factors for unintentional poisoning for 1–3 year olds are identified, so that appropriately targeted prevention measures for this age group can be implemented in NSW. However, as children aged 1–3 differ markedly in their level of poisoning risk from children aged <1 and age 4 years, unintentional poisoning risk factor studies that combine children aged 1–3 years with other ages may mask important findings for this three-year age group.

Historically, most studies of risk factors for unintentional injury, and unintentional poisoning in particular, have tended to combine all children aged 0–4 years of age. Yet, while studies since the 1950′s have shown age differences for unintentional poisoning risk in children aged 0–4 years [5,30], a review of international literature found that there was little research that attempted to differentiate risk factors within this age group. Although 56 unintentional poisoning risk factor studies have been conducted in developed countries, only six enrolled an age range younger than age 4 years [31]. One study enrolled children aged 0–2 years [15], four studies enrolled children aged 0–3 years [32-35] and one study enrolled children aged 3 years [36]. No studies were identified that enrolled only children aged 1–3 years. Furthermore, these six studies varied greatly in terms of the methodologies used and the risk factors examined.

First, they differed in their poisoning case definitions. Most definitions were very general, such as “the ingestion or inhalation of a substance which could be harmful”, the definition used by Beautrais et al. [34]. None excluded therapeutic errors by caregivers. This is important as the risk factors for unintentional poisonings in which children play a passive role due to the actions of other people [12,14] and those where children are actively involved in their self-poisoning are likely to be very different.

Second, three of the studies employed a case–control design and while this is an appropriate study design given the rare nature of unintentional poisoning events in young children, all three used hospital-based controls [32,33,35]. Hospital controls often represent a convenience sample [37], and can introduce sampling bias [37,38] as they may reflect caregivers who seek medical care for minor medical issues. As a result, hospital controls may not be representative of the population at risk for a poisoning event. Thus, the use of only hospital controls may have affected the risk factors identified by these three case–control studies.

Lastly, the studies looked at a limited range of risk factors. Current knowledge of contributing factors to injury in general highlights the importance of three behavioural factors- child compliance, caregiver supervision and the caregiver-child relationship as potential protective factors for children aged 1–3 years [39,40]. Only two studies reviewed included any of these risk factors [32,34] and none included all of them. Clearly, there is a need for further research on the range of risk factors for unintentional poisoning in the most vulnerable age group, children aged 1–3 years.

This study aimed to investigate the risk factors for unintentional poisoning in children aged 1–3 years and to address methodological issues identified with previous studies. These include employing a clear case definition and using appropriate methodologies to canvas a broad range of potential risk factors. In addition, this study aimed to employ a range of suitable control groups. Rather than using a hospital control group alone, this study aimed to enrol three age and sex-matched control groups: injured, sick and healthy children.

The three control groups were enrolled from two different populations, ED presentations (injured and sick) and the surrounding community (healthy). These three different groups were used to control for potential confounders and sampling bias associated with the use of a case–control study design [37]. The healthy control represented the background risk in children aged 1–3 years who did not experience an unintentional poisoning. The sick group controlled for potential confounders related to a child’s caregiver being predisposed to seek treatment at an emergency department. The injury group controlled for potential confounding variables related to a poisoning being a type of injury, such as caregiver behaviour or environmental hazards which could have led to any type of injury. The injury group also controlled for recall bias by caregivers stemming from guilt or regret and the use of socially appropriate responses. The study findings were considered more robust where more than one of the three case–control pairs demonstrated a significant difference for the same variable [37].

## Methods

### Study design and participants

This case–control study enrolled poisoning cases and injured and sick controls through the Sydney Children’s Hospital Emergency Department (SCHED) from 22 February 2005 to 14 January 2007. The Sydney Children’s Hospital is located in Randwick, NSW Australia and is one of the state’s three children’s tertiary care hospitals. A third control group, healthy controls, was enrolled into the study from 18 September 2005 to 31 October 2006 from the local community. Mothers were enrolled with their children into all four groups.

Poisoning cases were defined as children aged 1–3 years who presented to the SCHED for treatment of a poisoning after accessing a substance themselves (i.e., not given to them by a caregiver or other person). Injury and sick controls were defined as children aged 1–3 years who presented to the SCHED for treatment of an unintentional injury (other than poisoning) or an illness, respectively. Healthy controls were defined as children aged 1–3 years who attended a playgroup or a child care centre in the geographic areas served by the SCHED.

The sample size required for each of the four groups was 36 children aged 1–3 years. This was based on a required sample size of 35 for multiple regression with six predictors, an effect size of 0.5 for differences between groups in child temperament scores and parenting stress scores with 80% power and a level of significance of 5%. The sample size was increased to 36 to allow for equal numbers (6) in each single year of age and sex group (e.g., 6 males aged 1 year, 6 females aged 1 year).

The cases and three controls were to be matched by age (within 3 months) and sex to control for development-related aspects that may contribute to poisoning risk (e.g., physical growth) [14]. The South-eastern Sydney Area Health Service Research Ethics Committee approved the study.

### Procedures

#### Enrolment of emergency department cases and injury and sick controls

Children who met the poisoning case and injury and sick control group definitions were identified from all SCHED presentations on a weekly basis (excluding presentations ending in death). Letters explaining the study and inviting participation were sent to the residential address of mothers of a stratified sample (by child’s age and sex). Interested mothers were asked to contact the first author (MS). Letters were sent to 102 poisoning cases, 674 injury controls and 1014 sick controls.

Mother-child dyads were excluded if the mother reported any known developmental delay in her child or her child had any health conditions requiring long stays in hospital. Dyads were also excluded where the mother was not the primary caregiver, was unable to complete questionnaires in English, did not reply to the letter within 4 weeks or was not available to attend a 1.5 hour interview at the University of New South Wales which is located close to the SCHED.

Enrolment for each age-sex combination closed once six dyads had completed the interview and returned all study materials.

#### Enrolment of healthy community controls

Playgroups and child care centres in the geographic areas of interest were identified from lists of playgroups and centres registered with the NSW Playgroups Association and Australian Child Care Access Hotline, respectively. Playgroup coordinators and child care centre directors were asked to distribute study letters to mothers of eligible children (aged 1–3 years). A total of 2738 letters were sent to 67 child care centre directors for distribution. Playgroups NSW distributed approximately 1450–1740 study invitation letters to 29 playgroup coordinators (2 rounds of 25–30 letters/playgroup).

Interested mothers from both playgroups and child care centres contacted the first author. Healthy mother-child dyads underwent the same screening and group enrolment procedures used with the poisoning cases and injured and sick controls.

#### Interview

Mother-child dyads attended a three-part 1.5 hour digitally recorded appointment. First, the mother read and signed consent forms for herself and her child regarding participation in the study. Next, the child’s ability to perform a number of developmentally-related tasks was measured by the first author using the Denver Developmental Screening Test (DDST) [41]. Then the mother was asked to interact with her child during two 10 minute activities- a structured puzzle task (puzzle) and an unstructured free play session with toys in the room (free play). The first author was present in the room during the first task, but not the second. Finally, each mother was instructed to ask her child to pack away the toys (clean-up). The clean-up task was limited to three minutes. At the end of each appointment, each mother was given an envelope of questionnaires to complete and return.

### Measures

**Figure 1:**
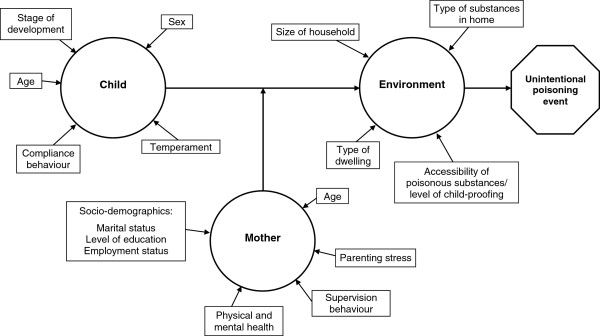
Schematic of risk factors investigated for unintentional poisoning in children aged 1–3 years.

The measures involved questionnaire, performance and observational methods and have been organised into the general domains of child, mother and environment (Figure 1).

#### Questionnaire – child domain

##### Demographics

Mothers completed the child section of the socio-demographic questionnaire (SDQ) which captured information on the following child variables: age (from date of birth), sex, number of hours in child care per week in own home and/or outside own home and total number of hours in child care per week.

##### Developmental level

The first author administered the Denver II (DDST) [41] to assess the current level of development of the child. This screening tool is a standardised instrument that measures the ability of children aged 0–6 years to perform developmentally-related tasks in four areas: personal-social (25 items), gross motor (39 items), fine motor-adaptive (29 items) and language (32 items). The child’s age at the time of the test (exact age) was calculated and the test was administered according to the test protocol.

Parental report was permitted for some items (as per test instructions). Each item attempted was scored as ‘pass’, ‘not pass (fail)’, ‘refused’ or ‘no opportunity’. For each item, the DDST documentation provided a value for the age at which 25, 50, 75 and 90 percent of the test group of children passed the item. The 90th percentile age value for the highest item passed was compared to the child’s age at the time of the test. The level of developmental ability was scored for each scale as ‘below expected’, ‘at expected’ or ‘above expected’.

##### Temperament

Mothers reported their child’s temperament using the Short Temperament Scale for Toddlers (STST). This questionnaire was developed through the Australian Temperament Project [42] and measures six factors reflecting temperament dimensions- approach, cooperation–manageability, persistence, rhythmicity, distractibility and reactivity. Mothers rated each item from 1–6, where 1 = ‘almost never’ and 6 = ‘almost always’. Six subscale scores and a composite easy/difficult score (average of approach, cooperation and reactivity scores) were calculated according to test instructions. Higher scores indicated the mother perceived her child as withdrawing (less approaching), uncooperative/unmanageable, not persistent, arrhythmic, non-distractible/non-soothable, highly reactive/irritable and difficult.

##### Compliance

Mothers reported their child’s ability to comply with maternal ‘do’ and ‘don’t do’ requests using a child compliance checklist (Ccomp). This checklist was adapted from a compliance checklist developed by Gralinski and Kopp [43] and measures the frequency of child compliance with parental requests. It comprises 31 items, with 15 items assessing the frequency of compliance with ‘do’ requests by parents (e.g., I have asked my child to put/pack his/her toys away) and 16 items assessing compliance with ‘don’t do’ requests (e.g., I have asked my child not to climb on furniture). Mothers rated their child’s frequency of compliance for each of the 31 items as ‘never complies’, ‘rarely complies’, ‘sometimes complies’, ‘often complies’, ‘always complies’ or ‘not applicable’ (i.e., I have never asked my child to do this). Maternal ratings were scored with the following numerical values: 1 = ‘never complies’, 2 = ‘rarely complies’, 3 = ‘sometimes complies’, 4 = ‘often complies’, 5 = ‘always complies’ and 9 = ‘non-applicable’. Ratings for the ‘do’ and ‘don’t do’ items were summed separately and in total. Scores for ‘do’ and ‘don’t do’ items were adjusted for ‘not applicable’ or missing answers. Higher total scores indicated the child complies more frequently with parental requests.

#### Questionnaire – mother domain

##### Demographics

Mothers completed the mother section of the SDQ which captured information on nine variables: age, marital status, highest level of education attained, country of birth, language spoken at home, employment status, number of working hours/week (if working outside the home), cigarette and alcohol use. Marital status was categorised as married/living with partner or single (i.e., never married, divorced). Highest level of education was categorised as a university level education or less attained or post university education.

##### Life events

Mothers completed a life events questionnaire adapted from the Holmes and Rahe Social Readjustment Rating Scale [44]. Mothers identified whether any of 30 listed life events occurred in their life in the year preceding study participation. The stress value weight(s) associated with each life event indicated were summed to identify a total life stress value.

##### Health status

Mothers rated their level of health on the General Health Questionnaire 28 (GHQ-28) [45]. The four GHQ-28 subscales were scored- somatic symptoms (A), anxiety (B), social dysfunction (C) and depression (D). The original numeric scores (1–4) were summed for each subscale using GHQ scoring. Answers of ‘3’ or ‘4’ (two-right hand columns) were scored ‘1’, whereas answers of ‘1’ or ‘2’ (other two columns) were scored ‘0’. These scores were summed for each variable and for a total test score. Higher scores indicated more of the attribute measured. A total sum of 5 or more indicated psychiatric ‘caseness’.

##### Parental stress

Mothers reported their level of parenting stress on the Parenting Stress Index- Short Form (PSI-SF) [46]. This questionnaire contains 36 items which correspond to three scales- Parental Distress (P_D) (12 items), Parent/Child Dysfunctional Interaction (PC_DI) (12 items) and Difficult Child (D_C) (12 items). Each item was scored from 1–5, where 1 = ‘strongly disagree’ and 5 = ‘strongly agree’. Variables scores and a total score were calculated according to test instructions. Higher scores indicate more of the attribute measured.

##### Supervision attributes

Mothers indicated their supervision attributes on the Parenting Supervision Attributes Profile Questionnaire (PSAPQ) [47]. The questionnaire comprised two parts. Part I included the following five scales: protectiveness (11 items), vigilance/proximity (6 items), worry (3 items), confidence (6 items) and values risk taking (4 items). Each item in Part 1 was scored from 1–5, where 1 = ‘strongly disagree’ and 5 = ‘strongly agree’. Part II consisted of three items: supervision during play activities (10 items), self-care (8 items) and risk activities (3 items). Each item in Part II was scored from 1–5 where 1 = ‘I’m often in another room and I go to my child when he/she calls me’ to 5 = ‘I’m often in the same room as my child and within arms reach’. When the statement was not applicable to the home or child, ‘NA’ was recorded. Scales in Part I and II were coded and summed according to test instructions. Higher scores indicate more of the attribute measured (Part I) and closer supervision during activities (Part II).

#### Questionnaire – environment domain

##### Socio-demographic data

Mothers indicated their post code, type of residence, number of bedrooms, ownership status and number and age of occupants on the environment section of the SDQ. The level of socio-economic disadvantage was derived using the Australian Socio-economic Index for Areas (SEIFA) [48] by postal code.

##### Poisons safety

Mothers indicated their poison safety practices for medicinal and non-medicinal (household) substances on the poison safety section of the SDQ. Poisoning storage questions assessed the height of usual storage of medicinal and household substances in different rooms and presence of child safety or other locks on usual places of storage. The number of accessible locations of medicinal and household substances in various rooms was derived (i.e. number of usual storage locations minus number of locations stored >= 1.4 metres or locked). The percent of total storage locations that were accessible was calculated for each room. Aspects of temporary storage for medicinal and household substances were also measured. Caregivers indicated if substances were intentionally stored in a temporary location and how often the substance was left out after use.

#### Observed mother-child interaction factors

Mother-child interaction factors were measured by applying the Parent–Child Interaction System (PARCHISY) [49,50] to the digital recordings of the puzzle and free play tasks. This observational coding system for mother-child interactions comprises 18 items; however, only five caregiver items (i.e., positive and negative control, positive and negative affect, responsiveness), five child items (positive and negative affect, responsiveness, independence, noncompliance) and three dyadic items (conflict, cooperation, reciprocity) were used. Each item was scored from 1 to 7 where 1 = ‘none of attribute shown’ to the 7 = ‘constant or exclusive use of the attribute measured’. The coding system was adapted to suit children aged 1–3 years and smaller coding intervals (i.e., 8 minutes total in 1 minute intervals). Medians of the eight 1-minute data points were used for the 13 PARCHISY variables scored for each task.

#### Observed child compliance

Children’s observed compliance with a maternal directive was measured by applying a compliance rating system employed by Kochanska et al. [51] to the digital recordings of the clean-up task. This compliance coding system used the following terms to describe child compliance: ‘committed compliance’, ‘situational compliance’, ‘passive noncompliance’, ‘overt resistance’, ‘defiance’ and ‘other’.

#### Reliability of coding

The authors, MS and AW, trained a person independent of the study to apply the PARCHISY and child compliance coding systems to the two mother-child interaction tasks and the child cleanup task, respectively. The coder was blinded to both the objectives of the study and study group membership. The observational data from the three tasks were copied to a DVD for each study dyad. Each DVD was labelled with a unique identifier and given to the coder in random order. The independent person coded all the DVDs initially and then re-coded a random selection of 10 percent of the DVDs. Intra-rater reliability for all three observation tasks was measured using Spearman’s rank correlations.

Intra-rater reliability for the puzzle task was 0.909 and 0.903 for the free play task. Across the two tasks, five individual items showed intra-rater reliability less than 0.6 and were excluded from further analysis (i.e., maternal positive control (free play), child positive affect (both tasks), child independence (puzzle), dyad cooperation (puzzle)). Intra-rater reliability for the observed compliance measure showed that all 10 DVDs matched on the rating assigned by the coder.

#### Circumstances of poisoning event

The socio-demographic questionnaire (SDQ) completed by mothers in the poisoning group included an ‘Event’ section. In this section, mothers provided a narrative regarding the circumstances of their child’s poisoning event and provided responses to a series of questions pertaining to the poisoning event. These questions assessed maternal perception of their child’s activities in accessing the substance as well as the substance type, use and storage. In addition, the questions assessed caregiver use of the NSW Poisons Information Centre, poisoning symptoms and actions taken upon presentation to hospital. The information collected was used for descriptive purposes and was not included in the analysis of factors predicting a poisoning event.

### Data analysis

This study was designed as a case–control study with one poisoning case matched to three separate controls (injury, sick and healthy) on age (within three months) and sex. However, some age-sex combinations could not be recruited into the study for the poisoning case group (i.e., females aged 1 and 3 years); therefore, cases and controls were not matched as planned. Instead, an unmatched analysis was done and the effects of age and sex were controlled in the analysis phase.

Data were analysed in case–control pairs - poisoning-injury (PI), poisoning-sick (PS) and poisoning-healthy (PH). Descriptive analyses were performed for variables by case–control pair. Univariate logistic regression analyses were conducted to assess the association between poisoning (outcome) and each independent variable (IV). A Likelihood ratio chi-square p-value of <0.20 was used to select IVs for the multivariate analyses.

Two IVs eligible for the multivariate models contained imputed values due to limited missing data. One variable in the PI multivariate model, PSI total score, contained an imputed value for one injury control. Data for one healthy control in the PH multivariate model contained an imputed value for two variables, PSI difficult child and PSI total score. Mean values of the PSI difficult child and PSI total score variables were imputed for the groups missing these values.

Multivariate logistic regression analyses were used to identify the predictors associated with a poisoning. Models were built interactively for each case–control pair with the child’s exact age (using date of birth at the time of the interview) and sex forced into each model. Forward selection was used in conjunction with two criteria to select IVs for the final model for each case–control pair - the lowest Akaike’s Information Criterion (AIC) with a Wald p-value of 0.1 or less. Correlations and multi-collinearity between IVs were checked before each new IV was added to the model. Where a new IV was highly correlated (>=0.5) or showed evidence of multi-collinearity (variance inflation factor >=2.5) with one or more IVs already in a model, the new IV was not added. Adjusted odds ratios (OR’s) and profile likelihood 95% CIs were estimated for all explanatory variables in the final model for each case–control pair. Model fits were assessed using the C-statistic. Analysis was performed using SAS V9.1.3 SP3.

## Results

### Overview

The study enrolled 10 poisoning cases and 113 controls- 40 injury, 37 sick and 36 healthy. Children who enrolled into the poisoning case and injury and sick control groups accounted for 10 percent, 6 percent and 4 percent, respectively, of children invited to participate in those groups. Children who enrolled into the healthy group accounted for less than one percent of all letters sent to playgroup coordinators and child care centre directors for distribution. Of the 67 child care centres and 29 playgroups that were asked to distribute study letters, 28 healthy controls came from 17 child care centres and the remaining eight controls came from seven playgroups.

Of the 123 study participants, one poisoning case and five controls were lost after enrolment for reasons including failure to return questionnaires (N=3), being unable to complete the interview session (N=1) and malfunction of the video recorder (N=2). The nine children remaining in the poisoning group comprised seven males and two females, with four children in the 1 and 2 years age groups and one child aged 3 years. Each of the control groups contained 36 children- 6 males and 6 females of age 1, 2 and 3 years. Table 1 presents demographic characteristics of the nine poisoning cases and the 108 injured, sick and healthy controls.

**Table 1:** Socio-demographic variables for poisoning cases and injury, sick and healthy controls

**Variable**	**Category**	**Case: Poisoning**	**Control 1: Injury**	**Control 2: Sick**	**Control 3: Healthy**
		**N (%) or**	**N (% ) or**	**N (%) or**	**N (%) or**
		**Median (Range)**	**Median (Range)**	**Median (Range)**	**Median (Range)**
**Child**					
Birth order	First	8 (89)	15 (42)	30 (83)	19 (53)
	Second	0 (0)	18 (50)	5 (14)	15 (42)
	Third or later	1 (11)	3 (8)	1 (3)	2 (6)
Number of hours in child care per week (both in and outside home)		16 (0–50)	15.5 (0–40)	18 (0–49)	24.5 (0–54)
**Mother**					
Age		37 (25–46)	36 (23–46)	35.5 (22–43)	38 (29–45)
Marital status	Married/living with partner	8 (89)	32 (89)	30 (83)	34 (94)
	Single (never married, separated, divorced)	1 (11)	4 (11)	6 (17)	2 (6)
Highest education level	Year 12 equivalent or below	0 (0)	6 (17)	4 (11)	4 (11)
	University degree or other technical qualifications	6 (67)	19 (53)	21 (58)	16 (44)
	Postgraduate studies	3 (33)	11 (31)	11 (31)	16 (44)
Country of birth	Australia	7 (78)	26 (72)	22 (61)	25 (69)
	Not Australia	2 (22)	10 (28)	14 (39)	11 (31)
Language spoken at home	English	9 (100)	36 (100)	35 (97)	34 (94)
	Not English			1 (3)	2 (6)
Employed outside home	Yes	7 (78)	20 (56)	22 (61)	32 (89)
	No	2 (22)	16 (44)	14 (39)	4 (11)
If employed outside home, mother works full-time (>35 hrs/wk)	Yes	2 (29)	3 (15)	4 (18)	4 (13)
	No	5 (71)	17 (85)	18 (82)	28 (88)
Smokes cigarettes	Yes	0 (0)	2 (6)	2 (6)	3 (8)
	No	9 (100)	34 (94)	34 (94)	33 (92)
Drinks alcohol	Yes	7 (78)	29 (81)	25 (69)	28 (82)
	No	2 (22)	7 (19)	11 (31)	6 (18)
**Environment**					
Occupant age structure	< 5 years	10 (0.33)	51 (0.38)	47 (0.39)	44 (0.34)
	5-9 years	1 (0.03)	16 (0.12)	4 (0.03)	9 (0.07)
	10-19 years	1 (0.03)	0 (0)	0 (0)	2 (0.02)
	20-39 years	10 (0.33)	50 (0.37)	46 (0.38)	36 (0.28)
	40-59 years	7 (0.23)	18 (0.13)	20 (0.17)	35 (0.27)
	60+ years	1 (0.03)	1 (0.01)	3 (0.03)	4 (0.03)
	Mean number of occupants (SD)	3.44 (0.73)	3.97 (0.77)	3.39 (0.77)	3.67 (0.79)
SEIFA Level of socio-economic disadvantage	Least disadvantaged	5 (56)	16 (44)	26 (72)	25 (69)
	4th	0 (0)	4 (11)	0 (0)	2 (6)
	3rd	3 (33)	11 (31)	9 (25)	5 (14)
	2nd	1 (11)	3 (8)	1 (3)	3 (8)
	Most disadvantaged	0 (0)	2 (6)	0 (0)	1 (3)
Residence type	Apartment	3 (33)	7 (19)	12 (33)	12 (33)
	Semi, terrace, townhouse, villa	3 (33)	13 (36)	7 (19)	10 (28)
	Stand-alone home	3 (33)	16 (44)	17 (47)	14 (39)

Table 2 presents characteristics of the poisoning events experienced by the nine children in the case group. Four cases were poisoned “while doing something [the mothers] didn’t know they could do yet”. When the event occurred, an adult was in the same room as the child in only one case, and in that one case, the mother was focused on something else at the time. Medicinal substances accounted for only four cases, but all three children who were admitted as an inpatient for treatment had ingested a medicinal substance. In three of the nine poisoning events, the substances accessed had been used in the previous 24 hours. In eight events, the substance was accessed less than 1.4 m from the floor. The substances involved in seven of the poisoning events were not in their usual place of storage when accessed. When the event was discovered, six caregivers called the NSW PIC prior to presenting to hospital.

**Table 2:** Characteristics of poisoning incidents

**Variable**	**Category**	**N (%)**
**Child demographics**		
Age	1 year	4 (44)
	2 years	4 (44)
	3 years	1 (11)
Sex	Male	7 (78)
	Female	2 (22)
**Developmental aspect of the event**		
Child was doing something the mother didn’t know the child could do yet	Yes	4 (44)
	No	5 (56)
Child was doing something the mother had previously told the child not to do	Yes	3 (33)
	No	6 (67)
Child was doing something the mother knew the child could do, but had never seen the child try to do before	Yes	2 (22)
	No	7 (78)
		
**Supervision preceding the event**		
Adult was in same room as child when the poisoning event occurred*	Yes	1 (11)
	No	8 (89)
**Substance type, use and storage**		
Type of substance accessed	Medicinal	4 (44)
	Non-medicinal	5 (56)
Type of packaging	Bottle with child-resistant cap	4 (44)
	Blister pack	1 (11)
	Other type of packaging	4 (56)
Substance used in last 24 hours	Yes	3 (33)
	No	5 (56)
	Not applicable	1 (11)
Substance accessed in location that was <1.4 m from ground*	Yes	8 (89)
	No	1 (11)
Substance was in its usual place of storage when accessed	Yes	2 (22)
	No	7 (77)
Mother felt that the usual place of storage for the substance accessed was inaccessible to child	Yes	5 (56)
	No	4 (44)
**Treatment**		
Caregiver called for advice prior to presenting to hospital	Yes, PIC	6 (67)
	Yes, other	2 (22)
	No	1 (11)
Child had symptoms associated with poisoning	Yes	3 (33)
	No	6 (67)
Patient disposition	Treated in ED and discharged	6 (67)
	Admitted for treatment as inpatient in ED	3 (33)

Note: * Abstracted from mothers’ description of the poisoning events.

### Univariate analyses

Univariate logistic regression analyses assessed the association between poisoning and each IV for each of the three case–control pairs (i.e., PI, PS, PH). Twenty-seven IVs met the 0.2 criteria to be included in the multivariate model for at least one case–control pair (Table 3). Variables from the child, mother and environment domains, as well as mother-child interaction variables, were associated with poisoning.

**Table 3:** Summary of univariate model results by domain

**Variable**	**Category**	**Case: Poisoning**	**Control 1: Injury**	**Control 2: Sick**	**Control 3: Healthy**
		**N (%) or**	**N (%) or**	**N (%) or**	**N (%) or**
		**Median (Range)**	**Median (Range)**	**Median (Range)**	**Median (Range)**
**Child**					
DDST Fine motor skills exceed expected level of development	Yes	6 (67)	29 (81)	**32 (89)**	**31 (86)**
	No	3 (33)	7 (19)	**4 (11)**	**5 (14)**
DDST Gross motor skills exceed expected level of development	Yes	4 (44)	**28 (78)**	**30 (83)**	**25 (69)**
	No	5 (56)	**8 (22)**	**6 (17)**	**11 (31)**
DDST Language skills exceed expected level of development	Yes	6 (67)	**32 (89)**	**33 (92)**	**32 (89)**
	No	3 (33)	**4 (11)**	**3 (8)**	**4 (11)**
Level of compliance*	Defensive responding	0 (0)	0 (0)	4 (11)	**3 (8)**
	Overt resistance	2 (22)	2 (6)	4 (11)	**2 (6)**
	Passive noncompliance	3 (33)	11 (31)	9 (25)	**5 (14)**
	Situational compliance	4 (44)	14 (39)	13 (36)	**14 (39)**
	Committed compliance	0 (0)	9 (25)	6 (17)	**12 (33)**
SDQ Number of hours in child care outside home		10.0 (0.0 - 28.0)	14.0 (0.0 - 30.0)	16.5 (0.0 - 46.0)	**20.5 (0.0 - 54.0)**
**Mother**					
GHQ Somatic symptoms (subscale A)		1.0 (0.0 - 4.0)	**0.0 (0.0 - 6.0)**	1.0 (0.0 - 6.0)	**0.0 (0.0 - 5.0)**
GHQ Anxiety (subscore B)		1.0 (0.0 - 6.0)	0.0 (0.0 - 7.0)	0.0 (0.0 - 7.0)	**0.0 (0.0 - 4.0)**
GHQ Total score		2.0 (0.0 - 13.0)	1.0 (0.0 - 17.0)	3.0 (0.0 - 21.0)	**0.5 (0.0 - 14.0)**
GHQ Psychiatric caseness (total score >5 indicating mental health issues)	Yes	4 (44)	**6 (17)**	13 (36)	**5 (14)**
	No	5 (56)	**30 (83)**	23 (64)	**31 (86)**
PSI Parental distress		22.0 (12.0 - 29.0)	**25.0 (15.0 - 43.0)**	**26.0 (13.0 - 49.0)**	**23.5 (12.0 - 44.0)**
PSI Parent–child dysfunctional interaction		13.0 (12.0 - 20.0)	**16.0 (12.0 - 28.0)**	14.0 (12.0 - 42.0)	**17.0 (12.0 - 27.0)**
PSI Difficult child		20.0 (12.0 - 35.0)	22.0 (13.0 - 52.0)	21.5 (13.0 - 54.0)	**25.0 (12.0 - 41.0)**
PSI Total score		58.0 (37.0 - 78.0)	**64.0 (44.0 - 100.0)**	**63.5 (38.0 - 133.0)**	**67.8 (39.0 - 102.0)**
PSAPQ Supervision during risk taking		4.0 (2.5 - 4.5)	**4.3 (2.0 - 5.0)**	4.0 (1.7 - 5.0)	4.0 (1.0 - 5.0)
**Environment**					
SDQ Number of children in household		1.0 (1.0 - 3.0)	**2.0 (1.0 - 3.0)**	1.0 (1.0 - 3.0)	2.0 (1.0 - 3.0)
SDQ Percent accessible of all medicinal substance storage locations in bathroom		0.0 (0.0 - 100.0)	0.0 (0.0 - 100.0)	**0.0 (0.0 - 100.0)**	0.0 (0.0 - 100.0)
SDQ Percent accessible of all medicinal substance storage locations in kitchen		0.0 (0.0 - 50.0)	**0.0 (0.0 - 100.0)**	0.0 (0.0 - 100.0)	0.0 (0.0 - 100.0)
SDQ Percent accessible of all household substance storage locations in other rooms (eat-in area, dining room, lounge room, family room)		0.0 (0.0 - 100.0)	0.0 (0.0 - 0.0)	**0.0 (0.0 - 100.0)**	**0.0 (0.0 - 100.0)**
SDQ Temporary storage: “When a medicinal substance is intentionally stored in a temporary location, how often is the substance left out after it is no longer needed?” **	None of the time	4 (80)	15 (63)	14 (59)	**12 (44)**
	Some of the time	1 (20)	7 (29)	8 (33)	**14 (52)**
	Most times	0 (0)	2 (8)	2 (8)	**1 (4)**
SDQ Temporary storage: “When a household substance is intentionally stored in a temporary location, how often is the substance left out after it is no longer needed?” **	None of the time	3 (75)	6 (55)	**5 (39)**	**2 (13)**
	Some of the time	1 (25)	4 (36)	**8 (62)**	**13 (81)**
	Most times	0 (0)	1 (9)	**0 (0)**	**1 (6)**
**Mother-child interaction**		**-**			
Maternal positive control during puzzle task		4.0 (3.0 - 5.0)	**3.0 (1.0 - 5.0)**	**3.0 (1.0 - 4.0)**	**3.0 (1.0 - 4.5)**
Maternal positive affect during puzzle task		1.5 (1.0 - 5.0)	**1.3 (1.0 - 4.0)**	**1.0 (1.0 - 3.0)**	1.0 (1.0 - 5.0)
Maternal positive affect during free play task		2.0 (1.0 - 5.0)	**1.5 (1.0 - 3.5)**	**1.3 (1.0 - 3.0)**	**1.0 (1.0 – 4.0)**
Child responsiveness during puzzle task		6.0 (3.5 - 7.0)	7.0 (1.0 - 7.0)	7.0 (2.0 - 7.0)	**7.0 (2.0 - 7.0)**
Child responsiveness during free play task		6.5 (3.0 - 7.0)	7.0 (2.0 - 7.0)	**7.0 (2.0 - 7.0)**	**7.0 (2.0 - 7.0)**
Child independence during free play task		6.0 (3.0 - 6.0)	**6.0 (3.5 - 7.0)**	**6.0 (3.0 - 7.0)**	6.0 (1.5 - 7.0)
Dyadic cooperation during free play task		6.0 (2.0 - 7.0)	6.8 (1.5 - 7.0)	**6.8 (2.0 - 7.0)**	7.0 (1.0 - 7.0)

Notes:

Values in bold indicate a likelihood ratio chi-square p-value of <0.20.

* The categories for this variable were aggregated into a binomial compliance measure for the univariate logistic regression. Children who exhibited ‘situational’ or ‘committed compliance’ to their mother’s clean-up request were considered compliant (Compliance= ‘Yes’). Children who exhibited ‘passive noncompliance’, ‘overt resistance’ or ‘defensive responding’ were considered noncompliant (Compliance = ‘No’).

** The responses for this variable were aggregated into a binomial temporary storage measure for the univariate logistic regression. The following responses were recoded to a ‘Yes’ response in the new binomial variable: ‘Some of the time’ or ‘Most times’. A ‘None of the time’ response was recoded to a ‘No’.

Five child domain variables were associated with poisoning for at least one case–control pair. Gross motor and language skill levels were eligible for all three multivariate models and fine motor skills level was eligible for the PS and PH multivariate models. The results indicate that poisoning cases were less advanced in their gross motor, fine motor and language skills than the three control groups. Poisoning cases were also less compliant and spent less time in care outside the home than healthy controls.

Nine mother domain variables were associated with poisoning for at least one case–control pair. Two variables, PSI parental distress and PSI total parenting stress, were eligible for all three multivariate models and three variables, GHQ somatic symptoms, GHQ psychiatric caseness and PSI parent–child dysfunctional interaction were all eligible for the PI and PH multivariate models. The GHQ results showed that a larger proportion of mothers of poisoning cases had mental health issues compared to mothers in all three control groups. Mothers of poisoned children reported lower levels of parenting stress than mothers in all three control groups for all PSI subscales (parental distress, parent–child dysfunctional interaction, difficult child) and PSI total score.

Six environment domain variables were associated with poisoning for at least one case–control pair. Two variables, percent of household substance storage locations accessible in other rooms and household substances left out after being temporarily stored, were eligible for two multivariate models- PS and PH. A larger percentage of mothers in the poisoning case group reported that household substances stored temporarily were not left out after use compared to all three control groups. However, the two temporary storage variables were excluded from the PS and PH multivariate models due to a large number of missing values (mothers indicated they never intentionally stored substances in temporary locations).

Seven mother-child interaction variables were associated with poisoning for at least one case–control pair. Two variables, PARCHISY maternal positive control (puzzle task) and maternal positive affect (free play) were eligible for all three models. Three variables were eligible for two of the three models- maternal positive affect during puzzle task (PI, PS), child responsiveness during the free play (PS, PH) and child independence during the free play (PI, PS). Mothers in the poisoning group exhibited more positive control and positive affect in both tasks than mothers in all three control groups.

### Multivariate analyses

#### Poisoning-injury model

Fourteen variables were included in the multivariate regression analyses for the PI pair (Table 3), along with the forced variables- child’s sex and exact age. The PSI parental distress variable was excluded from the model due to a correlation with child’s sex. No variables were excluded due to multi-collinearity with other model variables. The final model for the PI pair contained three IVs - use of positive control by the mother during the puzzle task, GHQ psychiatric caseness and mother’s level of supervision during risk taking activities. The max-rescaled RSquare for the overall model was 0.51 and 88.58 percent of pairs were concordant. The C-statistic indicated an excellent model fit at 0.89.

#### Poisoning-sick model

Thirteen variables were included in the multivariate regression analyses for the PS pair (Table 3), along with the forced variables- child’s sex and exact age. No IVs were excluded from the model due to multi-collinearity or correlation with other model variables. The final model for the PS pair contained two IVs - use of positive control by the mother during the puzzle task and the percentage of all medicinal substance storage locations in the bathroom that were accessible to young children (i.e., stored <1.4 m from ground and not lockable). The max-rescaled RSquare for the overall model was 0.55 and 89.81 percent of pairs were concordant. The C-statistic indicated an excellent model fit at 0.90.

#### Poisoning-healthy model

Eighteen variables were included in the multivariate regression analyses for the PH pair (Table 3), along with the forced variables- child’s sex and exact age. Two IVs, GHQ somatic symptoms and child responsiveness during the puzzle task, were excluded from the model due to correlations with GHQ psychiatric caseness and child’s exact age, respectively. No IVs were excluded from the model due to multi-collinearity with other model variables. The final model for the PH pair contained three IVs - use of positive control by the mother during the puzzle task, mother’s total score on the PSI and GHQ psychiatric caseness. The max-rescaled RSquare for the overall model was 0.54 and 89.2 percent of pairs were concordant. The C-statistic indicated an excellent model fit at 0.89.

### Comparison between models

**Figure 2:**
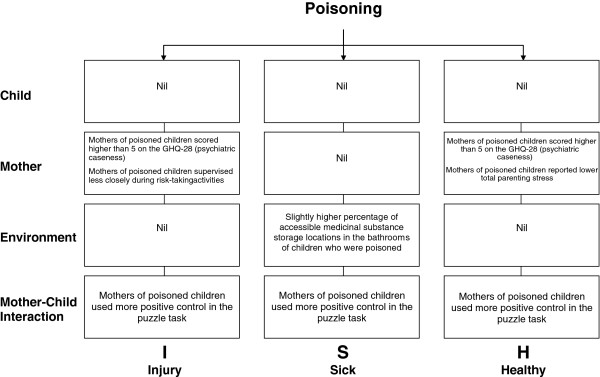
Risk factors for unintentional poisoning in final models.

Figure 2 presents a summary of the findings of the three final multivariate models by domain. Five child domain variables were included in the multivariate regression analyses for at least one case control pair. Of these variables, gross motor and language skills were assessed in all three models. However, none of the final models included a variable from this domain as a predictor for poisoning.

Only the PI and PH models included mother domain variables. Both models included GHQ psychiatric caseness as a significant predictor for poisoning. Children whose mothers scored higher than 5 on the GHQ-28 (using GHQ scoring) had higher odds of experiencing a poisoning compared to children whose mothers scored lower than 5 (PI model: OR: 36.54 (95%CI 2.25 - 999); PH model OR: 15.12 (95%CI 1.18 - 459.35)). For the PI model, children whose mothers supervised them more closely during risk taking activities had lower odds for a poisoning (PI model OR: 0.24 (95%CI 0.04 - 0.90)). For the PH model, children whose mothers reported higher parenting stress overall had lower odds of experiencing a poisoning (PH model OR: 0.90 (95%CI 0.80 - 0.98)).

Only the PS model included an environment domain variable, percentage of all medicinal substance storage locations in the bathroom that were accessible, as a predictor for poisoning. A one percent increase in accessible medicinal substance storage locations in the bathroom was associated with a slight increase in the odds of poisoning (PS model OR: 1.03 (95%CI 1.002 - 1.080)).

All three models included the same mother-child interaction variable. Children whose mothers used more positive control in their interactions during the puzzle task had higher odds of poisoning (PI model OR: 3.57 (95%CI 1.21 - 15.20); PS model OR: 20.03 (95%CI 3.01 - 375.01); PH model OR: 3.79 (95%CI 0.97 - 20.67)).

## Discussion

The current study investigated a broad range of potential risk factors for unintentional poisoning in children aged 1–3 years, including three behavioural factors- child compliance, caregiver supervision and the nature of mother-child interaction. The methodology employed appropriate questionnaire, performance and observational methods to explore these factors. In addition, the study excluded passive poisoning events (i.e., not due to self-access by the child) from the case group and controlled for potential confounders and sources of bias by including control groups from different populations.

The univariate analysis identified 27 factors that were associated with poisoning risk in one or more case–control pairs. These factors came from all four domains assessed (child, mother, environment and mother-child interaction) and six of the 27 factors were eligible for all three multivariate models. The final models included the following factors as significant predictors for unintentional poisoning in children aged 1–3 years: maternal use of positive control, parenting stress, GHQ psychiatric caseness, supervision during risk taking activities and accessible medicinal substance storage locations in the bathroom.

Mothers of cases used more positive control than mothers in all three control groups during the observation of mother-child interactions. The presence of this factor in all three final models adds weight to this finding; however, this finding could also be related to the age differences between the case and three control groups. Mothers of children who had been poisoned had younger children who may have needed more help than the older children in the control groups. Interestingly, the differences between the groups involved positive and encouraging controlling of children. There was little evidence of more negative control in the poisoning or any other groups.

The caregiver-child relationship plays an important role in helping children develop the ability to regulate their behaviour [23]. Research has shown that caregiver-child relationships characterised by a mutually responsive orientation can facilitate the process of self-regulation [52]. In particular, positive interactions that are mutually responsive have been demonstrated to result in better compliance and increase the likelihood that maternal rules are internalised by the child [52]. Child compliance and internalisation of maternal rules are two aspects of development that could potentially reduce a young child’s poisoning risk [39]. Mothers in the poisoning group also exhibited more positive affect in both tasks than mothers in the other groups. This difference may reflect maternal behaviour used to encourage young children to complete tasks which may be less necessary with older children. In addition, the children in the poisoning group showed signs of less responsiveness and less dyadic cooperation than children in the control groups. This may explain further the univariate finding regarding more positive affect in mothers of poisoning cases, but also may indicate the lack of a mutually responsive orientation which has implications for the development of child self-regulatory behaviour.

Close interactions between caregivers and children may also facilitate caregiver knowledge regarding their child’s development. This knowledge can help caregiver’s anticipate their child’s risk of exposure to poisons in their environment and implement appropriate prevention strategies [53]. Further research is warranted regarding the link between caregiver-child relationships, child self-regulation and unintentional poisoning risk.

Mothers in the poisoning group showed less parental stress than mothers in all three control groups. This measure of stress was not what might be predicted, particularly since the parental stress questionnaire was administered within 30 days of attendance at the Emergency Department for the poisoning event. It might be expected that a potentially life threatening event for their child would make caregivers more worried about their supervision skills; however, the results do not suggest this. Even if some of the caregivers had become more worried about parenting after the poisoning incident, their levels of parenting stress were still not as high as the control groups; parenting stress was high in the healthy group who had not had a recent episode relating to their child’s health and well-being.

In contrast, the mothers of children who had been poisoned had higher odds of being classified as psychologically distressed (based on the GHQ). This result is in line with a finding by Beautrais et al. [34]. Beautrais and colleagues showed that children of mothers who were prescribed anti-depressants and tranquilisers had higher rates of poisoning. In this study, mothers in the poisoning group may have other sources of distress in their lives such that they did not see parenting as a particular problem. It is also possible that mothers in the poisoning group experienced less parenting stress as almost all of the children in the case group were first-born and had no siblings.

The low level of parenting stress reported by mothers in the poisoning group may have indirectly contributed to their children’s unintentional poisoning as it is possible this reflects the caregivers’ approach to supervision and poisons storage practices (i.e., more relaxed). Almost all of the poisonings in the current study occurred when, in most cases, the substance was located less than 1.4 metres from the ground in a temporary storage place, and the caregiver was in another room. Inadequate poisons storage practices and supervision may also reflect inaccurate caregiver perception of the level of development [24,54]. The age of the case group and the circumstances of their poisoning events suggest that the caregivers were not yet aware that the children could or would try to access a substance stored in an unsafe manner.

Children in the poisoning group were supervised less closely during risk taking activities than children in the injury group. It is possible that this finding reflects supervision differences between boys and girls [54] as the poisoning group consisted mainly of boys. In addition to differences in how closely boys and girls are supervised, it may be that poisoning events occur when the supervision is not continuous, due to a distraction [34,55] or lapse in attention [56]. Descriptive data from eight of the nine poisoning events involving children in this study indicate that the poisoning events occurred when caregivers were not directly supervising their children (in another room) or were distracted.

The current study also explored the relationship between poisoning and a number of child developmental variables - fine and gross motor skills, language skills, compliance ability, positive and negative effect, independence, responsiveness and non-compliance. It found an association between poisoning and all of the developmental variables investigated except positive and negative effect for one or more case–control pairs. From the univariate analysis, children who were poisoned were more likely to score as less advanced in terms of fine and gross motor and language skills and be less compliant, less responsive and less independent in their behaviour compared to controls. In addition, gross motor and language skills were assessed using an age-standardised measure (based on the Denver II) and associated with poisoning for all three case–control pairs. Yet, none of the child developmental variables remained in the final models.

Age and sex differences were present between children in the case and three control groups in the study and it is possible that these differences may have influenced the factors identified as significant predictors of unintentional poisoning in children aged 1–3 years. Previous studies in NSW identified a link between age and sex and poisoning risk [4,14] and this link was attributed to developmental differences. As children of the same age and sex can differ markedly in terms of their level of development, the current study attempted to control for the confounding effect of these two variables by enrolling equal numbers of males and females into the case and control groups for each single year of age and then matching them by age and sex. However, the desired sample size and age-sex composition was only achieved for the three control groups; the nine children in the case group were younger than the controls and predominantly male. Consequently, the number of children in each age-sex group differed between the case and three control groups and matching could not be done; instead, the age and sex differences were controlled during the analysis. Thus, the smaller sample size and lack of matching could have implications for the interpretation of findings in this study as well as the ability of the study to identify predictors for unintentional poisoning in children aged 1–3 years, such as aspects of development that may increase poisoning risk.

Response rates were very low for all four study groups, but the number of children recruited into the poisoning group was very small. Even though the composition of the case group is consistent with the results from the population studies in NSW that showed that children aged 1–2 years and males have the highest rates of poisoning among children aged 0–4 years [4], unintentional poisoning presentations represented a much rarer occurrence compared to the number of children eligible for the control groups. The difficulty in enrolling cases and controls may reflect the type of enrolment procedure used. The current study was required to employ an ‘opt-in’ enrolment procedure to gain ethics approval, yet this enrolment approach has been linked to poor response rates [57,58]. In addition, the very low response rates in the control groups may have resulted in a potential source of bias. Compared to the three control groups, most mothers in the poisoning group had only one child, all had a university level degree or higher, a higher percentage worked full-time and none smoked. It is not known whether the observed socio-demographic differences reflect true differences between poisoning cases and other populations or were biases introduced as a result of the ‘opt-in’ enrolment procedure, which may have impacted results by affecting the types of participants who enrolled in the study [57]. Nonetheless, the presence of controls from different populations may have reduced the effect of potential bias in the study [37] as the univariate and multivariate analyses revealed the same finding for more than one model. Thus, while bias may have potentially affected the results in the study, the consistency of findings across the three models indicates that the effect of any such bias was minimal.

Overall, the results of this study draw attention to the importance of the caregiver-child relationship and caregiver influences in poisoning risk. Most children are walking well by age 14 months [23] allowing them to move about their environment. However, protective aspects of development, the cognitive ability to remember safety rules and the self-regulatory ability to stop themselves from accessing hazards, lag behind. Thus, physical development enables children to interact with hazards in their environment before they are able to understand the effect of their actions [59]. Given this imbalance, the caregiver-child-relationship may provide an avenue by which caregivers of children aged 1–3 years can raise their awareness of the rapid developmental changes occurring in their children. This information can then be used to reduce their children’s unintentional poisoning risk by implementing developmentally appropriate supervision and poisons storage practices to prevent exposure to hazardous substances.

This study could not be definitive, however, about the involvement of some of the caregiver factors due to the small number of poisoning cases and the age differences between cases and controls. Nevertheless, this study has helped to redefine the best approaches to study designs needed to understand more about caregiver involvement and the role of caregiver-child interactions in unintentional poisoning events. Larger samples of children who have experienced a poisoning are needed, along with control groups of younger children in order to avoid confounding of developmental differences.

## Conclusions

The current study indicates that maternal use of more positive control, accessible poison storage locations, less parenting stress and more psychological stress may contribute to unintentional poisoning. Less close supervision was also identified as a risk factor, suggesting that the proximity of supervision may be important for poison prevention. Further studies are needed, but caregiver education should focus on the benefits of close interaction with their child as a prevention measure as well as supervision and poisons storage practices appropriate for children aged 1–3 years.

## Competing interests

The authors declare that they have no competing interests.

## Authors’ contributions

MS conceived and designed the study, acquired and analysed the data and drafted the majority of the manuscript. AW assisted with the design of the study, coordinated the coding of observational data, assisted with the statistical analysis and interpretation of data, drafted portions of the manuscript and conducted a critical review of the manuscript for important intellectual content. DB assisted with the statistical analysis and interpretation of data and conducted a critical review of the manuscript for important intellectual content. LW assisted with interpretation of data and conducted a critical review of the manuscript for important intellectual content. All authors read and approved the final manuscript.

## Pre-publication history

The pre-publication history for this paper can be accessed here:

http://www.biomedcentral.com/1471-2431/13/88/prepub
